# Reformulation of Processed Yogurt and Breakfast Cereals over Time: A Scoping Review

**DOI:** 10.3390/ijerph20043322

**Published:** 2023-02-14

**Authors:** Sinead O’Mahony, Clare B. O’Donovan, Nuala Collins, Kevin Burke, Gerardine Doyle, Eileen R. Gibney

**Affiliations:** 1 Food Safety Authority of Ireland, The Exchange, Georges Dock, D01 P2V6 Dublin, Ireland; 2 Institute of Food and Health, School of Agriculture and Food Science, University College Dublin (UCD), Belfield, D04 V1W8 Dublin, Ireland; 3 School of Agriculture and Food Science, University College Dublin, Belfield, D04 V1W8 Dublin, Ireland; 4 Department of Mathematics and Statistics, University of Limerick, V94 T9PX Limerick, Ireland; 5 College of Business, University College Dublin, Belfield, D04 V1W8 Dublin, Ireland; 6 UCD Geary Institute of Public Policy, University College Dublin, Belfield, D04 V1W8 Dublin, Ireland

**Keywords:** reformulation, breakfast cereal, yogurt, retail food environment

## Abstract

Poor diet is responsible for a quarter of European non-communicable disease (NCD)-related deaths. The reformulation of sugar, salt, and saturated fat in processed packaged foods offers an opportunity to reduce consumption of nutrients of concern and also support a reduction in energy intake. To date, there have been no publications measuring progress in food reformulation by compiling published evidence for a food category. The aim of this scoping review was to identify, characterize and summarise the findings of studies analysing the reformulation of processed yogurt and breakfast cereals. The review answered the research question: “What is the impact of food reformulation on the nutrient quality of yogurt and breakfast cereals available in the retail environment?” The research protocol was defined based on PRISMA-ScR guidelines. Five databases were searched in May 2022. Thirteen studies, published between 2010 and 2021 and completed across seven countries were eligible for inclusion. There were sufficient eligible studies to identify trends in sodium, salt, and sugar reduction in breakfast cereals. However, there was minimal or no reduction in energy, which may bring into question the use of food reformulation as part of an overall health strategy for obesity reduction.

## 1. Introduction

A nutritional transition from unprocessed to processed foods and high calorie diets over the past 30 years is a recognised driver of the rising rates of overweight and obesity [1]. In 2016, 1.9 billion adults globally were reported to be living with overweight and obesity [2]. Poor diet is responsible for a quarter of European non-communicable disease (NCD)-related deaths [3]. Imbalanced (excessive) dietary intake of nutrients such as saturated fat, sugar, and salt is associated with an increased risk of NCDs, including coronary heart disease (CHD), stroke, type 2 diabetes, and cancer [4,5,6]. Evidence shows dietary improvement could reduce the rise in overweight and obesity, prevent one in five deaths globally, and a quarter of European deaths from NCDs [3,7].

Nutrients such as saturated fat, sugar, and salt, when consumed in high and unbalanced amounts contribute to NCDs, and are often the targets of public health recommendations, including food reformulation strategies [7]. In the context of NCDs, food reformulation is the reduction in energy and nutrients such as saturated fat, sugar, and salt in processed and packaged food in order to improve their nutrient quality and health profile [8]. 

Food reformulation has been described as a cost effective policy for the reduction of NCDs [9]. The World Health Organization (WHO) has recommended food companies reformulate their existing food products or introduce healthy alternatives in an effort to address the current obesity epidemic [10]. This advice has been heeded by national governments who have pursued food reformulation strategies such as the Public Health England (PHE) Sugar Reformulation Strategy, the Australian Healthy Food Partnership, and the Irish Roadmap for Food Product Reformulation [11,12,13]. 

There is evidence to suggest the reformulation of energy, saturated fat, sugar and salt in processed packaged foods has the potential to improve dietary intakes, health, and the healthiness of the food environment [8]. Despite this, observational studies report modest energy, sugar, saturated fat, and salt reduction in processed packaged foods in response to reformulation policy [14,15,16]. Reformulation progress is regularly monitored in detail by examining the food supply of a country or high-level comparison across countries [17,18]. To the best of our knowledge, there has been no investigation into the effect of reformulation policies on the nutrient content of food categories by compiling the available scientifically published evidence. Compiling available scientifically published evidence by food category could indicate progress in reformulation of food categories and yield lessons learned. 

The aim of this scoping review was to investigate progress in the reduction of energy, saturated fat, sugar, and salt in response to reformulation policies by compiling published scientific evidence. To achieve this, two food categories were chosen, namely, processed yogurts, particularly processed yogurt products with added ingredients and excluding natural yogurt made with milk and live yogurt cultures, and breakfast cereals [19]. Low fat and unsweetened yogurt and fortified breakfast cereals are recommended in the majority of food-based dietary guidelines globally and are recognized for their important contribution to essential nutrient intake in the diet [20]. Yogurt is recognized as a source of protein and calcium, contributing to many essential functions in the body such as bone and muscle health [21,22]. Breakfast cereals are a source of essential nutrients such as fibre, which is associated with numerous beneficial health effects including improved metabolic health [23,24]. Both breakfast cereals and yogurts are identified as food categories which make important contributions to the intake of essential micronutrients which are deficient in the diets of the European population, such as calcium from yogurt and folic acid from fortified breakfast cereals [19]. A study on yogurt consumption and nutrient intakes in America found yogurt contributed 21.7% of calcium intake in the adult population [25]. A study on the nutrient contribution of ready-to-eat breakfast cereals (RTEBC) amongst Irish adults found RTEBC contributed 10% of dietary fibre intake [26]. 

However, there is a great diversity of products in each of the food categories, ranging from products with little processing such as porridge oats and natural or classic yogurt, to products with high levels of sugar, salt, and saturated fat, such as granolas and flavoured yogurt made with the addition of sugar and/or cream [27,28]. The variation in the nutritional composition of products within these food categories offers an opportunity for reformulation to improve their nutrient profile. Indeed, these non-discretionary food categories are targeted by many reformulation programmes for the reduction of sugar and salt [12,13,29]. In Australia, yogurt was found to contribute 8.8% of free sugar intake in children 1–2 years and in the United Kingdom, yogurt contributed to 11.1% of free sugar intake in children 1–3 years [30,31]. In Ireland, breakfast cereals contributed 5.5% of sodium intake and 11% of free sugar intake in children 5–12 years [32]. Improving the nutritional composition of products with added sugar, fat, and salt within these two food categories could be an important way of contributing to improved nutrient intakes.

## 2. Materials and Methods

The study used a scoping review methodology, which is defined as an approach for mapping key concepts which define a research area [33]. A scoping review methodology was selected as the preferred approach given the heterogeneity in methodological approaches and reporting changes in food composition. The review was reported in line with the Preferred Reporting Items for Systematic Review and Meta-Analysis Extension for Scoping Review (PRISMA-ScR) guidelines [34]. A review protocol was drafted and followed the completion of this review. 

### 2.1. Research Question 

This review addressed the research question: “What is the impact of food reformulation policy on the nutrient quality of yogurt and breakfast cereals available in the retail environment?”

### 2.2. Defining Search String 

The review question was defined using the sample, phenomenon of interest, design, evaluation and research (SPIDER) framework [35] as outlined in Table 1. 

### 2.3. Systematic Search of the Relevant Literature 

The search terms, identified using the SPIDER framework, were developed into the following search strings: Reformulat* OR Reduc* OR Adapt* OR Lower* OR Less OR Reduc* OR Minimis* OR Modif* AND Sugar OR Salt OR Sodium OR Fat OR Saturated Fat OR Energy OR Kilocalories OR Kilojoules OR *calories AND “Nutrient Quality” OR “Nutrient Value” OR “Nutrition* Value” OR Healthiness OR Health* OR Nutrition OR “Healthy Eating” AND “Retail environment” OR “Food retailer” OR Shop OR Supermarket OR “Grocery store” OR “Retail outlet” OR Store OR Outlet OR Hypermarket OR Superstore OR “Cash and carry” AND Yoghurt OR Yogurt OR “Fromage frais” OR “Cultured dairy product” OR “Breakfast cereal” OR “Ready to eat breakfast cereal”. Multiple spellings of the term ‘yogurt’ were included to ensure a complete search. These search strings were applied in PubMed, Web of Science, EMBASE, Global Health (via OSPD) and Food Science and Technology Abstract in May 2022. A citation review of selected studies was undertaken to identify relevant studies from key papers. Finally, a hand search of papers was completed. 

### 2.4. Inclusion Criteria 

Scientific publications using a cross-sectional study design were considered for inclusion in this review. There was no limitation set on study date or geographical location. The population of interest was the general population. Food categories of interests were yogurt and breakfast cereals. The nutrients of interest were sugar, salt or sodium, and saturated fat, as well as energy. Of particular interest were studies which were completed using data collected from the retail food environment such as grocery stores and supermarkets. Nutrient quality was considered the nutritional value of the product. Food reformulation was considered as any effort to reduce or modify energy, fat, saturated fat, sugar, or salt content of the food categories of interest. Articles reported in English were included in this study. 

### 2.5. Appraisal of Studies for Review 

One reviewer defined and implemented the search strategy (S.O.) and imported the search findings into Endnote X9 for desktop. Duplicates were identified and removed using a two-step process, firstly by Endnote and then by one reviewer (S.O.). One reviewer (S.O.) performed title and abstract screening using Covidence. One reviewer (S.O.) performed review of all full texts (n = 65) and a second reviewer (C.B.O.) reviewed 50% (n = 33) of the full texts. Discrepancies were resolved by discussion at each stage. Where there was uncertainty or disagreement a third reviewer (E.R.G.) had the role of tie breaking. Reviewers were not blind to the journal titles or study author’s institutions.

### 2.6. Data Charting

A standardised and piloted form was used for data charting. Data was extracted for variables including study characteristics, data collection methodology, nutrient content at two time points and method of reporting. One reviewer (S.O.) completed data extraction from all included studies (n = 13) and a second reviewer (C.B.O.) completed extraction for 54% (n = 7) of included studies. Discrepancies were resolved by discussion and in cases were uncertainty or disagreement persisted, a third reviewer (E.R.G.) had the role of tie breaking.

### 2.7. Quality Assessment of Studies 

An assessment of study quality was undertaken using a tool developed for the critical appraisal of cross-sectional studies, AXIS [36]. One reviewer (S.O.) completed the study quality appraisal of all included studies (n = 13) and a second reviewer (C.B.O.) completed study quality appraisal for 54% (n = 7) of included studies. Discrepancies were resolved by discussion and when necessary, a third reviewer (E.R.G.) had the role of tie breaking. 

### 2.8. Data Synthesis and Analysis 

Results from each study was entered into Excel and the difference in nutrient content over time was calculated and/or recorded where it was specifically reported. A factor of 4.184 was used for the conversion of kilocalories to kilojoules [37]. Sodium and salt were reported as they were reported in the included studies. Tabular presentation of results and narrative analysis was completed for changes in energy, saturated fat, sugar, and salt or sodium in yogurts and breakfast cereals over time. A quantitative analysis of energy, sugar, saturated fat, salt, and sodium in breakfast cereals was completed using R Studio and presented in scatter graphs. This was not completed for yogurts due to the small number of papers identified in relation to nutrient changes in yogurt. 

## 3. Results

### 3.1. Search Findings 

**Figure 1:**
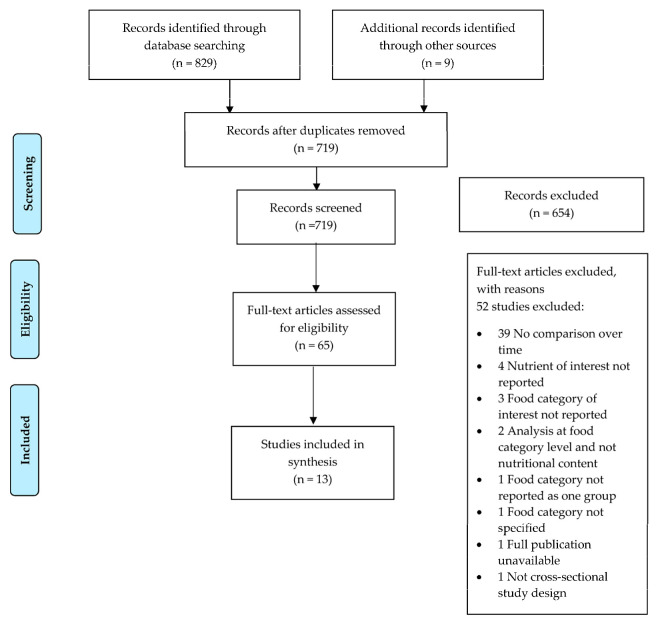
PRISMA Flow Diagram of Literature Review.

In total, 829 publications were retrieved from the five databases searched and nine studies were identified through a review of citations. After removing duplicates, 719 studies were included for review (PRISMA Flow Chart in Figure 1) [38]. Following abstract and title screening, 65 full-text articles were reviewed, with 13 articles included in the final review. Reasons for exclusion of full-text articles are outlined in Figure 1 and Supplementary Table S1. 

### 3.2. Study Characteristics 

The review found n = 10 studies reporting nutrient changes in breakfast cereals [27,39,40,41,42,43,44,45,46,47], n = 2 studies reporting nutrient changes in yogurt [15,48], and n = 1 reporting nutrient changes in both [49]. Eligible studies included data over a forty-year time period for breakfast cereals (1980–2020) and a fourteen-year time period for yogurts (2005–2019). All studies investigated breakfast cereals and/or yogurts targeting the general population. The thirteen studies included reported data collected across seven countries. Of the studies reporting on an individual country, five studies were based on the Australian food supply [39,40,44,47,48], two studies each reported on the UK food supply [15,41] and the New Zealand food supply [42,45], one study each reported on the Belgian [27], Chilean [49], Irish [46], and Canadian food supply [43]. All studies included (n = 13), used a cross-sectional study design to collect food product label information in store, online or using a combination of both [27,39,40,41,42,43,44,45,46,47,49]. A summary of study characteristics is outlined in Table 2.

### 3.3. Changes in Nutritional Content of Breakfast Cereal Products over Time

Of the thirteen studies included in this review, eleven examined changes in nutrient content of breakfast cereals over time [27,39,40,41,42,43,44,45,46,49]. The number of products included in these studies ranged from n = 10 [42] to n = 320 [27]. Six studies reported on paired breakfast cereals, meaning the same product was identified at both time points and compared across the timeframe [27,39,44,46,47,49]. Seven studies reported on two or more nutrients of interest and four studies reported on one nutrient—salt or sodium only [40,42,43,44]. A summary of studies reporting on nutrient change of breakfast cereals over time is outlined in Table 3. Supplementary Table S2 provides additional detail on the average nutrient content as well as the measure of variance as reported by each study. 

**Figure 2:**
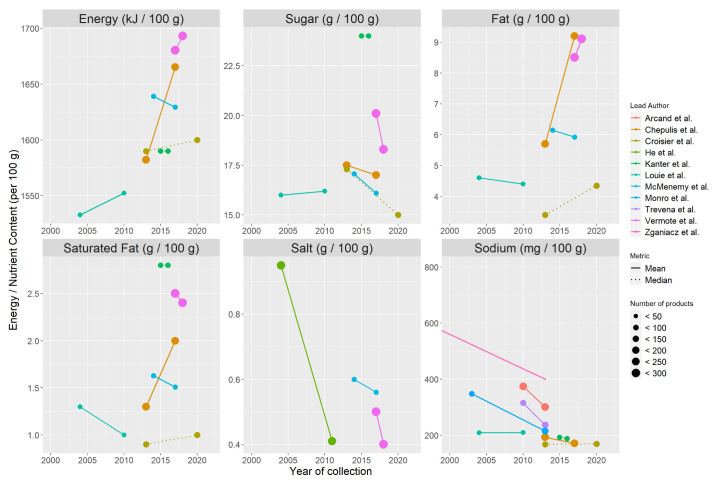
Energy (kJ) and nutrient changes in eligible studies (n = 11) investigating the reformulation of breakfast cereals over time [27,39,40,41,42,43,44,45,46,47,49].

Figure 2 outlines the trend in energy and nutrient changes over time per 100 g of breakfast cereals. In general, there was a trend towards an increase in energy (kJ) and total fat and a decrease in salt and sodium, sugar, and saturated fat. This is discussed in detail in the sections below. Notably, when mean sugar content decreased, total fat content increased in three of the studies [27,45,47]. 

### 3.4. Changes in Energy (kJ)

Six studies reported changes in the energy content in breakfast cereals per 100 g between 2004–2020 [18,27,39,46,47,49]. As outlined in Figure 2, there was a general trend towards an increase in energy content per 100 g of breakfast cereals in the included studies. As summarised in Table 3, three studies observed an increase in mean energy content of breakfast cereals, with the highest increase of 83.1 kJ per 100 g observed by Chepulis et al. in New Zealand. Croisier et al. observed a median increase in energy content of 10 kJ per 100 g [47]. Two of the six studies which reported energy content observed a slight decrease in energy content of breakfast cereals between 4.18–5.44 kJ per 100 g [27,46]. 

### 3.5. Changes in Salt and Sodium

Eleven eligible studies reported on changes in the salt or sodium content of breakfast cereals per 100 g between 1980–2020 [27,39,40,41,42,43,44,45,46,47,49]. As outlined in Figure 2, there was a strong trend towards salt and sodium reduction in breakfast cereals, with nine of the eleven studies included reporting a reduction in salt or sodium content of breakfast cereals. As detailed in Table 3, three studies reported mean salt reductions between 0.04–0.54 g per 100 g [27,41,46] and five studies reported mean sodium reductions between 21.7–406 mg per 100 g [40,42,43,44,45]. One study identified a median reduction in sodium content [49]. Two studies, both completed in Australia 10 years apart, identified an increase in salt and sodium content of breakfast cereals with a mean increase of 1.3 mg sodium between 2004 and 2010 [39], and a median increase of 2 mg per 100 g of breakfast cereals between 2013 and 2020 [47]. 

### 3.6. Changes in Sugar

Six studies reported changes in the sugar content of breakfast cereals per 100 g between 2004 and 202; this is detailed in Table 3 [27,39,45,46,47,49]. As outlined in Figure 2, four of the six studies reported a reduction in the sugar content of breakfast cereals with a mean sugar reduction between 0.5 and 1.8 g per 100 g [27,45,46] and median sugar reduction of 2.3 g per 100 g [47]. The highest mean sugar reduction of 1.8 g per 100 g was observed in Belgium [27]. Louie et al. identified a mean sugar increase of 0.2 g per 100 g in breakfast cereals in Australia, and Kanter et al. identified no change in median sugar content of breakfast cereals per 100 g in Chile [39,49]. 

### 3.7. Changes in Fat

Five studies reported changes in total fat content of breakfast cereals per 100 g between 2004 and 2020; this is detailed in Table 3 [27,39,45,46,47]. As outlined in Figure 2, three of the five studies reported an increase in total fat per 100 g in breakfast cereals. An increase in mean fat content of 0.6–3.5 g per 100 g [27,45] and a median increase of 0.95 g per 100 g was observed [47]. The largest mean total fat increase of 3.5 g per 100 g was observed in New Zealand [45]. A slight decrease of 0.2 g per 100 g in breakfast cereal was observed in two studies [39,46]. 

### 3.8. Changes in Saturated Fat

Six studies reported changes in the saturated fat content of breakfast cereals per 100 g between 2004 and 2020, which is detailed in Table 3 [18,27,39,46,47]. As shown in Figure 2, four studies observed a reduction in mean saturated fat content between 0.1 and 0.3 g per 100 g [27,39,46]. Three studies observed no change or an increase in mean or median saturated fat content [45,47,49]. The largest increase was 0.7 g per 100 g observed in New Zealand [45]. 

### 3.9. Changes in Nutritional Content of Yogurt Products over Time

Of the thirteen studies included in this review, three examined the reformulation of yogurt products. All three studies had a focus on the change in sugar content of yogurts over time, between 2010 and 2020 [15,48,49]. The number of products included in these studies ranged from n = 38 [49] to n = 898 [15]. Moore et al. and Kanter et al. found a reduction in median sugar content in sugar-sweetened yogurts of 1.5 g/100 g and 2.6 g/100 g, respectively [15,49]. Moore et al. also found a mean reduction in the energy and sugar content of paired yogurts which were on the market at both time points. Walker et al. reported an increase across energy and all nutrients over time except salt, which saw no change [48]. A summary of reported changes of the nutrient content in yogurts over time is outlined in Table 4. Supplementary Table S2 provides additional detail on the average nutrient content as well as the measure of variance as reported by each study.

### 3.10. Study Quality 

Study quality assessment was undertaken using Appraisal Tool for Cross-Sectional Studies (AXIS) [36]. The quality of the included studies was fair; this is summarized in Supplementary Table S3. No study was excluded due to issues identified in study quality. 

## 4. Discussion

The aim of this scoping review was to investigate progress in the reduction of energy, saturated fat, sugar, and salt in yogurts and breakfast cereals as a result of food reformulation by compiling published scientific evidence. To the best of our knowledge, there has been no investigation on the effect of food reformulation on the nutrient content of food categories by compiling the available published evidence. Compiling available scientifically published data for food categories could help identify progress and trends in the reformulation of food categories and yield lessons learned. The review found n = 10 studies reporting nutrient changes in breakfast cereals, n = 2 studies reporting nutrient changes in yogurt and n = 1 reporting nutrient changes on both. Similar trends in the increase or decrease in energy and nutrient changes were seen across eligible studies. There was a strong trend towards a decrease in salt and sodium content, but no change or an increase in the energy content of breakfast cereals. There was a limited number of publications on yogurts, meaning trends in nutrient changes were inconclusive. 

### 4.1. Success in Sodium and Salt Reformulation 

It is estimated that approximately 99% of the world’s adult population has a mean salt intake above the recommended levels (less than 5 g/day) which can lead to increased risk of CVD and high blood pressure [50]. To address this, numerous governments and policy makers have pursued food reformulation initiatives to reduce the salt content in processed packaged foods [51]. The effects of these policies are evident in the results of this review which found salt and sodium content of breakfast cereals reduced overall in seven countries [27,40,41,42,43,46,49]. However, Croisier et al. found that reductions in the sodium content of breakfast cereals reported in earlier studies in Australia (between 2003 and 2013) [40,44] had stabilized or reversed between 2013 and 2020 [47]. This finding suggests that reformulation efforts may plateau over time and constant monitoring is needed. This stabilization could be due to a number of reasons, such as technological challenges in food processing, consumer acceptance and competing demand for reformulation of other nutrients such as sugar [52,53]. Regardless, this finding suggests that in order for reformulation gains to be maintained continued monitoring and industry engagement is required.

### 4.2. Limited Progress in Energy Reduction 

Alongside targeting specific nutrients such as salt and sugar, food reformulation is often considered as a policy to support the reduction of population overweight and obesity. However, this review found an increase (or no change) in breakfast cereal mean or median energy content in all but one study [46], suggesting nutrient changes are occurring with no reduction in energy. This finding is of concern in relation to the effectiveness of food reformulation for the purpose of reducing rates of population overweight and obesity, which is not possible without energy intake reduction. This has been noted before, where Gressier et al. previously reported that food reformulation was unlikely to result in a reduction of energy intake in the diet [7]. This finding indicates that policy makers should be cautious in describing food reformulation as a policy for the reduction of overweight and obesity where energy reduction is not a mandatory requirement. 

Despite this, the current review did observe energy reduction in yogurts and breakfast cereals in matched pairs compared over time points, suggesting that methodological approaches can influence the interpretation of findings in this area [15,27]. Food reformulation is defined as the reduction of the nutrient content of a product, and does not encompass product range diversification such as the introduction of ‘reduced’ versions of the same product [7]. Thus, measuring nutrient reduction in overall category average can dilute the true effect of food reformulation efforts as product portfolio diversification can result in product reformulation making little difference to the average nutrient content of a food category. This finding requires further investigation as it has implications for how food reformulation is monitored and reported. 

### 4.3. Methodological Consideratins in Reformulaiton Monitoring and Reporting 

To help address methodological variation, in 2011, the Global Food Monitoring Initiative published a protocol to compare and monitor the nutritional composition of branded foods and later the INFORMAS collaboration published a protocol outlining a standard for the collection of food composition data [54,55]. Despite the publication of the global standards over ten years ago, this study found a broad range of methods used to collect, organise, and analyse food composition data. Studies included in this review used different data collection methods, had different sample sizes of food products included in a category, reported results in different ways (mean and median) over varying time intervals and by food category average and product pairs. This heterogeneity in data collection, analysis and reporting presents a challenge for study comparison. There were fewer studies available for yogurt than breakfast cereals and the lack of harmonisation in the data collection methods, product categorisation and analysis and reporting made drawing conclusions for yogurts more challenging given the fewer number of studies. Continued efforts in the harmonization of food composition data collection and monitoring are required to support comparison and trends in food composition over time.

### 4.4. Reformulation Policy Type and Progress 

Food and beverage reformulation is usually prompted by national policy or law [7]. However, the effectiveness of voluntary policies and initiatives, such as industry-led reformulation policies, to improve the healthiness of food environments has been called into question due to their weak accountability systems to measure and ensure the achievement of targets [56,57,58]. The majority of studies included in this review reported on voluntary food reformulation policies such as those implemented in Australia, New Zealand, and the UK [15,39,45]. These programmes appeared to lead to a reduction in the nutrient of interest, such as sugar [59], but did not result in a change in the overall nutrient profile of the food. A mandatory policy of food warning labels introduced in Chile in 2016, and reported by Kanter et al. found a decrease in the sugar content of yogurts but no change in breakfast cereals [49]. 

### 4.5. Reformulation of Single or Multipe Nutrients 

Introduction of front-of-pack nutrition labelling (FoPNL) using an evaluative nutrient profile has been described as a lever for food reformulation [60]. The literature reports a positive effect of FoPNL on the reduction of restricted nutrients in processed food [14,60,61,62,63]. Kanter et al. and Vermote et al. observed reformulation in anticipation of mandatory and voluntary FoPNL schemes, respectively [27,49]. Reformulation in anticipation or response to the introduction of FoPNL can result in reductions across energy and nutrients of concern as observed by Vermote et al. [27]. In contrast, reformulation programmes which target individual nutrients alone may result in an increase in another nutrient. This is well described in the study by Gressier et al. who found that a third of studies reporting on single nutrient reductions also reported increases in other nutrients including fat and salt [64]. This phenomenon was also observed in this review in the form of a sugar–fat seesaw. The sugar–fat seesaw is a term used to describe a reciprocal relationship between the intake of sugars and fat which has been previously reported from observational data [65]. A systematic review confirmed the existence of the sugar–fat seesaw on a percentage energy basis, which could possibly be explained by a combination of mathematical and food compositional effects [66]. This review observed a trend towards an increase in total fat content where there was a decrease in sugar content per 100 g of breakfast cereals [27,45,47]. In order for food reformulation programmes to achieve the intended effect of population dietary improvement, overall food composition needs to be considered and monitored. 

### 4.6. Review Strengths and Limitaitons 

The strength of this review is that it is the first to investigate the effect of food reformulation on energy and nutrients of public health concern in particular food categories by compiling published scientific literature. Data from eligible studies were from different time periods, countries, and nutrients, and there was little discrepancy between reviewers. The review allows conclusions to be drawn on different nutrients in particular food categories. The review also has limitations; it only focused on the reformulation of a small range of food categories: yogurts and breakfast cereals. Although this provides an in-depth insight into the effect of food reformulation policies, it is a narrow view, and so may not be representative of the effect of food reformulation policies on other food categories or across the food environment in general. Some of the studies included had small sample sizes which means they may not be an accurate representation of the market at that time and may introduce bias. There was a limited number of studies available for yogurts, meaning it was not possible to draw conclusions for this food category. Further studies are needed to fill the gaps identified by this review as there is missing evidence about the effect of reformulation policy on changes in individual food category nutrient content and profile over time.

## 5. Conclusions

In conclusion, the contribution of this review is that it is possible to monitor trends in nutrient changes in a food category in response to nutrition policy, such as reformulation, using published scientific evidence, where there is sufficient published evidence available to compile. This finding could be important for policy makers when deciding on methods to review food reformulation progress. It was not possible to draw clear conclusions for processed yogurts as there were few eligible studies, indicating a need for additional monitoring and publishing on the nutrient composition of yogurt over time. The eligible studies included in this review used heterogenous data collection, categorization and reporting within food categories, making comparison challenging. This finding indicates there is a need for further work to harmonise how food composition information is collected and reported. A trend in the reduction of sodium and a trend towards sugar reduction in breakfast cereals was clear in eligible studies, which if translated into consumer purchasing habits, could result in dietary improvement. Despite this, only one study saw a reduction in energy content of breakfast cereals. The reduction of sugar, saturated fat and sodium in population diets could reduce the prevalence of chronic diseases such as coronary heart disease and stroke. However, food reformulation of single nutrients without energy reduction will not contribute to a reduction in rates of overweight and obesity. This calls into question the use of food reformulation as a tool for reducing overweight and obesity. More studies on the effect of food reformulation policies on energy content of prepackaged foods are required.

## Figures and Tables

**Table 1:** SPIDER (Sample, Phenomenon of Interest, Design, Evaluation and Research Type) table for the selection of studies.

Sample	Phenomenon of Interest	Design	Evaluation	Research Type
Yogurt and breakfast cereals sold in retail food environment	Reformulation (voluntary or mandatory) of energy, saturated fat, sugar, salt	All study designs	Change in energy, saturated fat, sugar, or salt content	Quantitative

**Table 2:** Characteristics of 13 studies investigating the reformulation of breakfast cereals and yogurts included in this review.

Lead Author	Year of Publication	Study Design	Food Category	Nutritional Characteristics Reported	Target Population of Food Products Considered	Time Frame	Country	Reformulation Programme or Policy Lever Discussed
Walker et al. [48]	2010	Cross-sectional survey of one supermarket.	Yogurt	Energy (kJ), total fat, saturated fat, sugar, and sodium	General population	2005 vs 2008	Australia	None
Louie et al. [39]	2012	Cross-sectional survey of one supermarket in 2004 and two supermarkets in 2010.	Breakfast cereal	Energy (kJ), total fat, saturated fat, sugars, and sodium	General population	2004 vs 2010	Australia	Voluntary—Australian Food and Grocery Council introduced a Daily Intake Guide Heart Foundation Tick programme.
Trevena et al. [40]	2014	Cross-sectional survey of four supermarkets.	Breakfast cereal	Sodium	General population	2010 vs 2013	Australia	Voluntary—The Australian Food and Health Dialogue.
He et al. [41]	2014	Cross-sectional survey of six supermarkets.	Breakfast cereal	Salt	General population	2004 vs 2011	UK	Voluntary—UK Salt Reduction Programme.
Monro et al. [42]	2015	Cross-sectional survey of four supermarkets in 2003 and corresponding data from 2013 obtained from branded food database.	Breakfast cereal	Sodium	General population	2003 vs 2013	New Zealand	Voluntary—Heart Foundation Tick programme.
Arcand et al. [43]	2016	Cross-sectional survey of four supermarkets.	Breakfast cereal	Sodium	General population	2010 vs 2013	Canada	Voluntary—Health Canada’s sodium reduction.
Zganiacz et al. [44]	2017	Cross-sectional survey in supermarkets.	Breakfast cereal	Sodium	General population	1980 vs 2013	Australia	None.
Chepulis et al. [45]	2017	Cross-sectional survey in two supermarkets.	Breakfast cereal	Energy (kJ), total fat, saturated fat, sugar, and sodium	General population	2013 vs 2017	New Zealand	Introduction of FSANZ—NPSC in 2016 for mandatory restriction of nutrition and health claims made on food.
Kanter et al. [49]	2019	Cross-sectional survey of five supermarkets.	Breakfast Cereal and Yogurt	Energy (kcal), saturated fat, sugar, and sodium	General population	2015 vs 2016	Chile	Mandatory—Chile’s Law of Food Labelling and Advertising, 2016.
Moore et al. [15]	2020	Cross-sectional survey of five supermarkets.	Yogurt	Sugar and Energy (kcal) for paired products	General population	2016 vs 2019	UK	Voluntary—PHE Sugar Reduction Programme.
Vermote et al. [27]	2020	Cross-sectional survey of seven supermarkets.	Breakfast cereal	Energy (kcal), total fat, saturated fat, sugar, and salt	General population	2017 vs 2018	Belgium	Voluntary—Introduction of FoPNL NutriScore.
McMenemy et al. [46]	2020	Cross-sectional survey of six supermarkets in 2014 and seven supermarkets in 2017.	Breakfast cereal	Energy (kcal), fat, saturated fat, sugar, and salt	General population	2014 vs 2017	Ireland	Voluntary—Salt reformulation programme, EC Selected Nutrient Initiatives.
Croisier et al. [47]	2021	Cross-sectional survey of four supermarkets.	Breakfast cereal	Energy (kJ), fat, saturated fat, sugar, and sodium	General population	2013 vs 2020	Australia	Voluntary—Healthy Food Partnership.

kJ = kilojoule, kcal = kilocalorie, EU = European Union, FSANZ = Food Standards Australia New Zealand, NPSC = Nutrient Profiling Scoring Criterion, FoPNL = Front-of-Pack Nutrition Label, EC = European Commission, PHE = Public Health England (now referred to as the Office for Health Improvement and Disparities, (OHID)), UK = United Kingdom.

**Table 3:** Energy (kJ) and nutrient changes in the 11 studies investigating the reformulation of breakfast cereal over time.

Lead Author	Time Interval between Data Collections (Years)	Number of Breakfast Cereals Identified	Energy (kJ)/ 100 g	Sugar (g)/ 100 g	Fat (g)/ 100 g	Saturated Fat (g)/ 100 g	Salt (g)/ 100 g	Sodium (mg)/ 100 g
Louie et al. [39]	6	2004 n = 67 2010 n = 67	+19.6 ^y^	+0.2 ^y^	−0.2 ^y^	−0.3 ^y^	-	+1.3 ^y^
Trevena et al. [40]	3	2010 n = 125 2013 n= 159	-	-	-	-	-	−79 ^y^
He et al. [41]	7	2004 n = 306 2011 n = 290	-	-	-	-	−0.54 ^y^	-
Monro et al. [42]	10	2003 n = 109 2013 n = 176	-	-	-	-	-	−133 ^y^
Arcand et al. [43]	3	2010 n = 230 2013 n = 250	-	-	-	-	-	−74 ^y^
Zganiacz et al. [44]	33	1980 n = 10 2013 n = 10	-	-	-	-	-	−406 ^y^
Chepulis et al. [45]	4	2013 n = 247 2017 n = 243	+83 ^y^	−0.5 ^y^	+3.5 ^y^	+0.7 ^y^	-	−21.7 ^y^
Kanter et al. [49] ^##^	1	2015 n = 93 2016 n = 93	NC ^y^	NC ^y^	-	NC ^y^	-	−4 ^#^
Vermote et al. [27]	1	2017 n = 320 2018 n = 330 Pd n = 275	+12.97 ^y^ −4.18 ^y^	−1.8 ^y^ −1 ^y^	+0.6 ^y^ −0.2 ^y^	−0.1 ^y^ −0.1 ^y^	−0.1 ^y^ −0.1 ^y^	- -
McMenemy et al. [46]	3	2014 n = 86 2017 n = 86	−5.44 ^y^	−0.96 ^y^	−0.23 ^y^	−0.12 ^y^	−0.04 ^y^	-
Croisier et al. [47]	7	2013 n = 34 2020 n = 134	+10 ^#^	−2.3 ^#^	+0.95 ^#^	+0.1 ^#^	-	2 ^#^

^#^ = reported using median value, ^y^ = reported as mean value, ‘-’ = not reported, NC = no change, ^##^ = grouping included breakfast cereals and bars, Pd = paired breakfast cereals at time point 1 and time point 2.

**Table 4:** Energy (kJ) and nutrient changes in the three studies investigating the reformulation of yogurt over time.

Lead Author	Time Interval between Data Collections (Years)	Number of Yogurt Products Identified	Energy (kJ)/100 g	Sugar (g)/100 g	Fat (g)/100 g	Saturated Fat (g)/100 g	Sodium (mg)/100 g
Walker et al. [48]	3	2005 n = 169 2008 n = 90	+30 ^#^	+0.2 ^#^	+1.6 ^#^	+1 ^#^	NC ^#^
Kanter et al. [49]	1	2015 n = 38 2016 n = 38	NC ^#^	−2.6 ^#^	-	-	-
Moore et al. [15]	3	2016 n = 898 2019 n = 893 Pd n = 539	- Pd −10.6 ^y^	−1.5 g ^#^ Pd −0.65 ^y^	- Pd NC ^y^	- -	- -

^#^ = reported using median value, ^y^ = reported using mean value, ‘-’ = not reported, NC = no change Pd = paired yogurts at time point 1 and time point 2.

## Data Availability

The data presented in this study are available in Supplementary Table S2.

## Supplementary Materials

The following supporting information can be downloaded at: https://www.mdpi.com/article/10.3390/ijerph20043322/s1, Table S1: Average nutrition information as declared in the review-eligible study, Table S2: Studies reviewed in full and not eligible for inclusion and reason for exclusion, Table S3: Quality assessment of 13 included studies using Appraisal Tool for Cross-Sectional Studies (AXIS).
