# Supplementary material for: Reformulation of Processed Yogurt and Breakfast Cereals over Time: A Scoping Review
Source: Int J Environ Res Public Health. 2023 Feb 14;20(4):3322. doi: 10.3390/ijerph20043322 (PMC9964677; doi:10.3390/ijerph20043322)
Supplement: Supplementary file 1 [file ijerph-20-03322-s001.zip › Supplementary Table S1_January 2023.pdf]

Supplementary Table S1. Average nutrition information as declared in the review eligible study.

Lead Author	Year of Publication	Reporting method	Food category	Time Point 1	Number of products	Energy per 100g				Fat per 100g		Saturated Fat per 100g		Sugar p
						kJ	Variance	Kcal	Variance	g	Variance	g	Variance	g
Walker et al.	2010	Median (IQR)	Yogurt	2005	169	385	140			1	3	0.6	2	13.7
Louie et al.	2012	Mean (Standard Error)	Breakfast cereal	2004	67	1532.7	18.9			4.6	0.5	1.3	0.2	16
Trevena et al.	2014	Mean (Standard Deviation)	Breakfast cereal	2010	125									
He et al.	2014	Mean (Standard Deviation)	Breakfast cereal	2004	306									
Monro et al.	2015	Mean (Standard Deviation)	Breakfast cereal	2003	109									
Arcand et al.	2016	Mean (Standard Deviation)	Breakfast cereal	2010	230									
Zganiacz et al.	2017	Mean (Standard Deviation)	Breakfast cereal	1980	10									
Chepulis et al.	2017	Mean (Standard Deviation)	Breakfast Cereal	2013	247	1582.1	153.4			5.7	5	1.3	1.4	17.5
Kanter et al.	2019	Median (min-max)	Breakfast cereal	2015	93			380	362-403			2.8	1.2-4.6	24
			Yogurt (normal)	2015	38			90	78-93					13.7
Moore et al.	2020	Median (min-max)	Yogurt	2016	898									11.9
Vermote et al.	2020	Mean (Standard Error)	Breakfast cereal	2017	320			401.6	2	8.5	0.3	2.5	0.1	20.1
McMenemy et al.	2020	Mean (Standard Deviation)	Breakfast cereal	2014	86	1639.17	145.2	387.2	36.91	6.15	6.49	1.63	1.91	17.06
Croisier et al.	2021	Median (min-max)	Breakfast cereal	2013	134	1590	1530-1630			3.4	1.6-8.4	0.9	0.4-1.5	17.3

er 100g	Salt per 100g		Sodium per 100g		**Time Point 2**	**Number of products**	Energy per 100g				Fat per 100g		Saturated Fat per 100g		Sugar per 100g		Salt per 100g		Sodium per 100g	
Variance	g	Variance	mg	Variance			kJ	Variance	Kcal	Variance	g	Variance	g	Variance	g	Variance	g	Variance	mg	Variance
6.7			59	23	2008	90	418	151			2.6	2	1.6	2	13.9	5			58	21
1.4			209	29.7	2010	67	1552.3	14.9			4.4	0.5	1	0.1	16.2	1.4			210.3	28.6
			316	NA	2013	159													237	NA
	0.95	0.74			2011	290											0.41	0.39		
			348	275	2013	176													215	183
			375	246	2013	250													301	242
			806	NA	2013	10													400	NA
10			193.3	187.9	2017	243	1665.2	194.2			9.2	9.8	2	2.3	17	9.7			171.6	161.3
16.5-30			192	81-320	2016	93			380	362-403			2.8	1.2-4.5	24	16.5-30			188	81-290
8.2-14.5					2016	38			90	65-97					11.1	8.2-13.1				
8.8,13.6					2019	893									10.4	6.6,13				
0.5	0.5	0			2018	330			404.7	2	9.1	0.4	2.4	0.1	18.3	0.5	0.4	0		
9.98	0.6	0.36			2017	86	1629.34	132.28	385.9	32.3	5.92	6	1.51	1.79	16.1	9.62	0.56	0.34		
7.9-24.8			167.5	23-355	2020	134	1600	1540-1640			4.35	1.8-8.8	1	0.5-1.6	15	7.1-22.3			169.5	16-300
