# Supplementary material for: Reformulation of Processed Yogurt and Breakfast Cereals over Time: A Scoping Review
Source: Int J Environ Res Public Health. 2023 Feb 14;20(4):3322. doi: 10.3390/ijerph20043322 (PMC9964677; doi:10.3390/ijerph20043322)
Supplement: Supplementary file 1 [file ijerph-20-03322-s001.zip › Supplementary Table S2_January 2023.pdf]

Published Year	Title	Reason for Exclusion
2021	The sugar content of foods in the UK by category and company: A repeated cross-sectional study, 2015-2018	Nutrient of interest no report
2020	Trends in Free Sugar Content of Slovenian Pre-Packaged Foods and Non-Alcoholic Beverages	Nutrient of interest no report
2017	Do Brands Serve as Reliable Signals of Nutritional Quality? The Case of Breakfast Cereals	Nutrient of interest no report
2019	Variations in Sugar Content of Flavored Milks and Yogurts: A Cross-Sectional Study across 3 Countries	No comparison over time
2020	Sugar Content and Nutritional Quality of Child Orientated Ready to Eat Cereals and Yoghurts in the UK and Latin America; Does Food Policy Matter?	No comparison over time
2020	The nutritional content of children's breakfast cereals: a cross-sectional analysis of New Zealand, Australia, the UK, Canada and the USA	No comparison over time
2019	Tracking kids food: Comparing the nutritional value and marketing appeals of child-targeted supermarket products over time	Food category of interest not reported
2008	The National Food and Nutrient Analysis Program: A decade of progress	Food category of interest not reported
2010	Nutritional quality and labelling of ready-to-eat breakfast cereals: the contribution of the French observatory of food quality	No comparison over time
2014	A comparative study of the sodium content and calories from sugar in toddler foods sold in low- and high-income New York City supermarkets	No comparison over time
2007	Choosing breakfast: how well does packet information on Australian breakfast cereals, bars and drinks reflect recommendations?	No comparison over time
2004	Trans, saturated, and unsaturated fat in foods in the United States prior to mandatory trans-fat labeling	No comparison over time
2018	UK children's breakfast cereals - an oral health perspective	No comparison over time
2012	Exploring the landscape of nutrition related marketing in Canada: is it guiding consumers to more healthful dietary patterns?	No comparison over time
2022	Nutritional quality and degree of processing of childrens foods assessment on the french market	No comparison over time
2021	Changes in the Use of Non-nutritive Sweeteners in the Chilean Food and Beverage Supply After the Implementation of the Food Labeling and Advertising Law	Nutrient of interest no report
2021	Claims on Ready-to-Eat Cereals: Are Those With Claims Healthier?	No comparison over time
2019	Availability and interpretation of the labeling of dairy products in Montevideo- Uruguay: transversal study on declarations of nutritional properties related to energy and total fats	No comparison over time
2019	Nutrient profiling and food prices: what is the cost of choosing healthier products?	No comparison over time
2014	Nutritional quality, labelling and promotion of breakfast cereals on the New Zealand market	No comparison over time
2022	Nutritional quality of wholegrain cereal-based products sold on the Italian market: data from the FLIP study	No comparison over time
2016	Investigating nutrient profiling and Health Star Ratings on core dairy products in Australia	No comparison over time
2017	The healthfulness and prominence of sugar in child-targeted breakfast cereals in Canada	No comparison over time
2021	A Cross-Sectional Audit of Nutrition and Health Claims on Dairy Yoghurts in Supermarkets of the Illawarra Region of New South Wales, Australia	No comparison over time
2018	Evaluating the Nutritional Content of Children's Breakfast Cereals in Australia	No comparison over time
2019	An Audit of the Nutrition and Health Claims on Breakfast Cereals in Supermarkets in the Illawarra Region of Australia	No comparison over time
2017	Nutrient Content in Yoghurt: A Comprehensive Survey of the UK Yoghurt Market in Advance of Government Mandated Sugar Reduction and Reformulation	No comparison over time
2017	Assessment of the construct validity of the Australian Health Star Rating: a nutrient profiling diagnostic accuracy study	No comparison over time
2015	Comparative Performance of NEMS-S Surveys in Latino Corner Stores in the Greater Boston Area	Analysis at food category level and not nutritional content;
2022	Encouraging healthier grocery purchases online: A randomised controlled trial and lessons learned	No comparison over time
2009	Healthy food choices and physical activity opportunities in two contrasting Alabama cities	No comparison over time
2018	Child- and adult-orientated breakfast cereals: a cross sectional analysis of nutrient profile	No comparison over time
2020	A survey on salt content labeling of the processed food available in Malaysia	No comparison over time
2016	The nutritional quality of foods carrying health-related claims in Germany, The Netherlands, Spain, Slovenia and the United Kingdom	No comparison over time
2017	Consumption of ultra-processed foods predicts diet quality in Canada	No comparison over time
2017	Use of Added Sugars Instead of Total Sugars May Improve the Capacity of the Health Star Rating System to Discriminate between Core and Discretionary Foods	No comparison over time
2019	Does point-of-sale nutrition information improve the nutritional quality of food choices?	No comparison over time
1997	Nutrition labelling of sugar-containing foods in 1996 compared with 1989	Full publication Unavailable
2021	Which companies dominate the packaged food supply of New Zealand and how healthy are their products?	No comparison over time
2020	The holistico-reductionist Siga classification according to the degree of food processing: an evaluation of ultra-processed foods in French supermarkets	No comparison over time
2021	Fermented foods: availability, cost, ingredients, nutritional content and on-pack claims	No comparison over time
2018	Evaluation of the nutrient content of yogurts: a comprehensive survey of yogurt products in the major UK supermarkets	No comparison over time
2021	Evaluating the nutritional quality of UK meat and dairy analogues compared to conventional animal products using multiple nutrient profiling models	No comparison over time
2018	Evaluating the <10:1 wholegrain criterion in identifying nutrient quality and health implications of UK breads and breakfast cereals	No comparison over time
2021	A comparison of four different nutritional profile models in their scoring of critical nutrient levels in food products targeted at Brazilian children	No comparison over time
2021	Breakfast cereals carrying fibre-related claims: do they have a better nutritional composition than those without such claims? Results from the food labelling of Italian products (FLIP) study	No comparison over time
2009	Availability of more healthful food alternatives in traditional, convenience, and nontraditional types of food stores in two rural Texas counties	Analysis at food category level and not nutritional content;
2016	Analysis of critical nutrient levels (sugar, fat, and salt) declared in processed foods marketed in Loja-Ecuador	Food category not specified
2013	Amount of sugar in Australian breakfast cereals is not associated with energy density or glycaemic index: results of a systematic survey	No comparison over time
2018	Food reformulation and nutritional quality of food consumption: an analysis based on households panel data in France	Nutrient of interest not reported;
2019	Prevalence of child-directed marketing on breakfast cereal packages before and after Chile's food marketing law: a pre- and post-quantitative content analysis	Food category not reported as one group;
2021	Sugars, Salt, Saturated Fat and Fibre Purchased through Packaged Food and Soft Drinks in Europe 2015–2018: Are We Making Progress?	Study design not cross-sectional
