# Supplementary material for: Reformulation of Processed Yogurt and Breakfast Cereals over Time: A Scoping Review
Source: Int J Environ Res Public Health. 2023 Feb 14;20(4):3322. doi: 10.3390/ijerph20043322 (PMC9964677; doi:10.3390/ijerph20043322)
Supplement: Supplementary file 1 [file ijerph-20-03322-s001.zip › Supplementary Table S3 Axis Quality Appraisal_January 2023.pdf]

Supplementary Table S3. Quality assessment of 13 included studies using Appraisal Tool for Cross-Sectional Studies (AXIS).

**Question**	Walker et al.	Louie et al.	Trevena et al.	He et al.	Monro et al.	Arcand et al.	Zganiacz et al.	Chepulis et al.	Kanter et al.	Moore et al.	Vermote et al.	McMenemy et al.	Croisier et al.
Were the aims/objectives of the study clear	Y	Y	Y	Y	Y	Y	Y	Y	Y	Y	Y	Y	Y
Was the study design appropriate for the stated aim(s)?	Y	Y	Y	Y	Y	Y	Y	Y	Y	Y	Y	Y	Y
Was the sample size justified?	Y	Y	Y	Y	Y	Y	N	Y	Y	Y	Y	N	Y
Was the target/reference population clearly defined? (is it clear who the research was about)	Y	Y	Y	Y	Y	Y	Y	Y	Y	Y	Y	Y	Y
Was the sample frame taken from an appropriate population base so that it closely represented the target/reference population under investigation?	Y	Y	Y	Y	Y	Y	N	Y	Y	Y	Y	Y	Y
Was the selection process likely to select subjects/participants that were representative of the target / reference population under investigation?	Y	Y	Y	Y	Y	Y	N	Y	N	Y	Y	Y	Y
Were measures undertaken to address and categories non responders?	N	N	N	N	N	N	N	CT	CT	N	N	N	o
Were the risk factor and outcome variables measured appropriate to the aims of the study?	Y	Y	Y	Y	Y	Y	Y	Y	Y	Y	Y	Y	Y
Were the risk factor and outcome variables measured correctly using instruments / measurements that had been trialed, piloted and published previously?	Y	Y	Y	Y	Y	Y	Y	Y	Y	Y	Y	Y	Y
Is it clear what was used to determine statistical significance and / or precision estimates? (e.g. p-values, confidence intervals)	Y	Y	Y	N	Y	Y	Y	Y	N	Y	Y	N	Y
Were the methods (including statistical methods) sufficiently described to enable them to be repeated?	Y	Y	Y	N	Y	Y	Y	Y	Y	Y	Y	N	Y
Were the basic data adequately described?	Y	Y	Y	Y	Y	Y	Y	Y	Y	Y	Y	Y	Y
Does the response rate raise concerns about non - response bias?	N	N	N	N	N	N	N	N	CT	N	N	N	N
If appropriate, was information about non responders described?	N	N	N	N	N	N	N	CT	CT	CT	Y	N	N
Were the results internally consistent?	Y	Y	Y	Y	Y	Y	Y	Y	Y	Y	Y	Y	Y
Were the results presented for all the analyses described in the methods?	Y	Y	Y	N	Y	Y	Y	Y	Y	Y	Y	Y	Y
Were the authors' discussions and conclusions justified by the results?	Y	Y	Y	Y	Y	Y	Y	Y	Y	Y	Y	Y	Y
Were the limitations of the study discussed?	N	Y	Y	N	Y	Y	N	N	Y	Y	Y	Y	Y
Were there any funding sources or conflicts of interest that may affect the authors' interpretations of the results?	N	CT	N	N	N	N	N	N	N	N	Y	CT	N
Was ethical approval or consent of participants attained?	N	Y	N	N	CT	CT	N	CT	CT	N	CT	N	N

Y= yes, N = N, CT = Can't Tell
